# 2-(5-Cyclo­hexyl-3-isopropyl­sulfanyl-1-benzofuran-2-yl)acetic acid

**DOI:** 10.1107/S1600536811055152

**Published:** 2012-01-14

**Authors:** Hong Dae Choi, Pil Ja Seo, Uk Lee

**Affiliations:** a Department of Chemistry, Dongeui University, San 24 Kaya-dong Busanjin-gu, Busan 614-714, Republic of Korea; b Department of Chemistry, Pukyong National University, 599-1 Daeyeon 3-dong, Nam-gu, Busan 608-737, Republic of Korea

## Abstract

In the title compound, C_19_H_24_O_3_S, the cylohexyl ring adopts a chair conformation. In the crystal, molecules are linked *via* pairs of O—H⋯O hydrogen bonds, forming inversion dimers. These dimers are further stabilized by weak inter­molecular C—H⋯π inter­actions, and by slipped π–π inter­actions between the furan rings of adjacent mol­ecules [centroid–centroid distance = 3.557 (2) Å, inter­planar distance = 3.301 (2) Å and slippage = 1.325 (2) Å].

## Related literature

For the biological activity of benzofuran compounds, see: Aslam *et al.* (2009 ▶); Galal *et al.* (2009 ▶); Khan *et al.* (2005 ▶). For natural products with benzofuran rings, see: Akgul & Anil (2003 ▶); Soekamto *et al.* (2003 ▶). For the crystal structure of related compound, see: Seo *et al.* (2011 ▶).
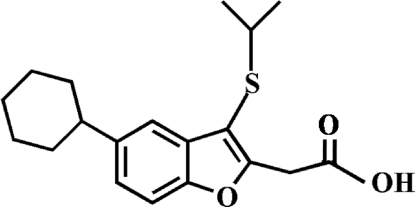


## Experimental

### 

#### Crystal data

C_19_H_24_O_3_S
*M* *_r_* = 332.44
Triclinic, 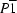

*a* = 9.1261 (2) Å
*b* = 9.5308 (2) Å
*c* = 10.5577 (2) Å
α = 72.228 (1)°
β = 79.702 (1)°
γ = 85.724 (1)°
*V* = 860.19 (3) Å^3^
*Z* = 2
Mo *K*α radiation
μ = 0.20 mm^−1^
*T* = 173 K
0.36 × 0.27 × 0.23 mm

#### Data collection

Bruker SMART APEXII CCD diffractometer
Absorption correction: multi-scan (*SADABS*; Bruker, 2009 ▶) *T*_min_ = 0.931, *T*_max_ = 0.955
16313 measured reflections
4297 independent reflections
3683 reflections with *I* > 2σ(*I*)
*R*_int_ = 0.028

#### Refinement

*R*[*F* ^2^ > 2σ(*F* ^2^)] = 0.036
*wR*(*F* ^2^) = 0.098
*S* = 1.05
4297 reflections
214 parameters
H atoms treated by a mixture of independent and constrained refinement
Δρ_max_ = 0.33 e Å^−3^
Δρ_min_ = −0.24 e Å^−3^

### 

Data collection: *APEX2* (Bruker, 2009 ▶); cell refinement: *SAINT* (Bruker, 2009 ▶); data reduction: *SAINT*; program(s) used to solve structure: *SHELXS97* (Sheldrick, 2008 ▶); program(s) used to refine structure: *SHELXL97* (Sheldrick, 2008 ▶); molecular graphics: *ORTEP-3* (Farrugia, 1997 ▶) and *DIAMOND* (Brandenburg, 1998 ▶); software used to prepare material for publication: *SHELXL97*.

## Supplementary Material

Crystal structure: contains datablock(s) global, I. DOI: 10.1107/S1600536811055152/ff2049sup1.cif

Structure factors: contains datablock(s) I. DOI: 10.1107/S1600536811055152/ff2049Isup2.hkl

Supplementary material file. DOI: 10.1107/S1600536811055152/ff2049Isup3.cml

Additional supplementary materials:  crystallographic information; 3D view; checkCIF report

## Figures and Tables

**Table 1:** Hydrogen-bond geometry (Å, °) *Cg*1 is the centroid of the C2–C7 benzene ring.

*D*—H⋯*A*	*D*—H	H⋯*A*	*D*⋯*A*	*D*—H⋯*A*
O3—H3*O*⋯O2^i^	0.87 (2)	1.81 (2)	2.6745 (14)	179 (2)
C15—H15*B*⋯*Cg*1^ii^	0.99	2.77	3.578 (2)	140

Symmetry codes: (i) 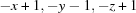; (ii) 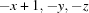.

**Fig. 1.**
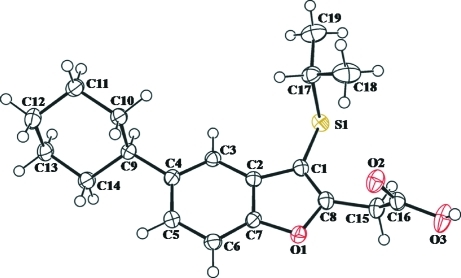
The molecular structure of the title compound with the atom numbering scheme. Displacement ellipsoids are drawn at the 50% probability level. H atoms are presented as small spheres of arbitrary radius.

**Fig. 2.**
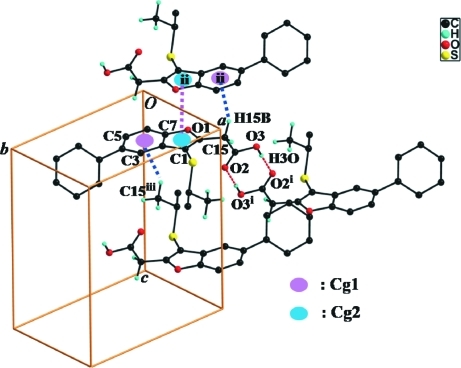
A view of the O—H···O, C—H···π and π–π interactions (dotted lines) in the crystal structure of the title compound. H atoms non-participating in hydrogen-bonding were omitted for clarity. [Symmetry codes: (i) - x + 1, - y - 1, - z + 1; (ii) - x + 1, - y, - z; (iii) -x + 1, - y, - z + 1.]

## supplementary crystallographic information

### Comment

Substituted benzofuran derivatives have drawn much interest owing to their valuable biological properties such as antibacterial and antifungal, antitumor and antiviral, and antimicrobial activities (Aslam *et al.*, 2009, Galal *et al.*, 2009, Khan *et al.*, 2005). These benzofuran derivatives occur in a wide range of natural products (Akgul & Anil, 2003; Soekamto *et al.*, 2003). As a part of our ongoing study of 2-(5-cyclohexyl-1-benzofuran-2-yl)acetic acid derivatives containing 3-methylsulfanyl (Seo *et al.*, 2011) substituent, we report herein the crystal structure of the title compound.

In the title molecule (Fig. 1), the benzofuran unit is essentially planar, with a mean deviation of 0.032 (1) Å from the least-squares plane defined by the nine constituent atoms. The cyclohexyl ring has the chair conformation. In the crystal structure, the carboxyl groups are involved in intermolecular O—H···O hydrogen bonds (see, Fig. 2 & Table 1), which link the molecules into centrosymmetric dimers. These dimers are further stabilized by weak intermolecular C—H···π interactions (see, Fig. 2 & Table 1; Cg1 is the centroid of the C2–C7 benzene ring). Additionally, the crystal packing (Fig. 2) shows a weak slipped between the furan rings of adjacent molecules, with a Cg2···Cg2^ii^ distance of 3.557 (2) Å and an interplanar distance of 3.301 (2) Å resulting in a slippage of 1.325 (2) Å (Cg2 is the centroid of the C1/C2/C7/O1/C8 furan ring).

### Experimental

Ethyl 2-(5-cyclohexyl-3-isopropylsulfanyl-1-benzofuran-2-yl)acetate (396 mg, 1.1 mmol) was added to a solution of potassium hydroxide (336 mg, 6 mmol) in water (10 ml) and methanol (15 ml), and the mixture was refluxed for 5h, then cooled. Water was added, and the solution was extracted with dichloromethane. The aqueous layer was acidified to pH=1 with concentrated hydrochloric acid and then extracted with chloroform. The organic layer was separated, dried over magnesium sulfate, filtered and concentrated at reduced pressure. The residue was purified by column chromatography (ethyl acetate) to afford the title compound as a colorless solid [yield 84%, m.p. 423–424 K; *R*_f_ = 0.55 (ethyl acetate)]. Single crystals suitable for X-ray diffraction were prepared by slow evaporation of a solution of the title compound in acetone at room temperature.

### Refinement

H atom in the carboxy group is found in a different Fourier map and refined freely. The other H atoms of C atoms were positioned geometrically and refined using ariding model, with C—H = 0.95 Å for aryl, 1.00 Å for methine, 0.99 Å for methylene and 0.98Å for methyl H atoms, respectively. *U*_iso_(H) =1.2*U*_eq_(C) for aryl, methine and methylene, and 1.5*U*_eq_(C) for methyl H atoms.

### Crystal data

**Table:**

C_19_H_24_O_3_S	*Z* = 2
*M**_r_* = 332.44	*F*(000) = 356
Triclinic, *P*1	*D*_x_ = 1.284 Mg m^−^^3^
Hall symbol: -P 1	Mo *K*α radiation, λ = 0.71073 Å
*a* = 9.1261 (2) Å	Cell parameters from 6099 reflections
*b* = 9.5308 (2) Å	θ = 2.6–28.4°
*c* = 10.5577 (2) Å	µ = 0.20 mm^−^^1^
α = 72.228 (1)°	*T* = 173 K
β = 79.702 (1)°	Block, colourless
γ = 85.724 (1)°	0.36 × 0.27 × 0.23 mm
*V* = 860.19 (3) Å^3^	

### Data collection

**Table:**

Bruker SMART APEXII CCD diffractometer	4297 independent reflections
Radiation source: rotating anode	3683 reflections with *I* > 2σ(*I*)
graphite multilayer	*R*_int_ = 0.028
Detector resolution: 10.0 pixels mm^-1^	θ_max_ = 28.4°, θ_min_ = 2.1°
φ and ω scans	*h* = −12→12
Absorption correction: multi-scan (*SADABS*; Bruker, 2009)	*k* = −12→12
*T*_min_ = 0.931, *T*_max_ = 0.955	*l* = −14→14
16313 measured reflections	

### Refinement

**Table:**

Refinement on *F*^2^	Primary atom site location: structure-invariant direct methods
Least-squares matrix: full	Secondary atom site location: difference Fourier map
*R*[*F*^2^ > 2σ(*F*^2^)] = 0.036	Hydrogen site location: difference Fourier map
*wR*(*F*^2^) = 0.098	H atoms treated by a mixture of independent and constrained refinement
*S* = 1.05	*w* = 1/[σ^2^(*F*_o_^2^) + (0.0473*P*)^2^ + 0.2514*P*] where *P* = (*F*_o_^2^ + 2*F*_c_^2^)/3
4297 reflections	(Δ/σ)_max_ < 0.001
214 parameters	Δρ_max_ = 0.33 e Å^−^^3^
0 restraints	Δρ_min_ = −0.24 e Å^−^^3^

### Special details

**Table:**

Geometry. All esds (except the esd in the dihedral angle between two l.s. planes) are estimated using the full covariance matrix. The cell esds are taken into account individually in the estimation of esds in distances, angles and torsion angles; correlations between esds in cell parameters are only used when they are defined by crystal symmetry. An approximate (isotropic) treatment of cell esds is used for estimating esds involving l.s. planes.
Refinement. Refinement of F^2^ against ALL reflections. The weighted R-factor wR and goodness of fit S are based on F^2^, conventional R-factors R are based on F, with F set to zero for negative F^2^. The threshold expression of F^2^ > 2sigma(F^2^) is used only for calculating R-factors(gt) etc. and is not relevant to the choice of reflections for refinement. R-factors based on F^2^ are statistically about twice as large as those based on F, and R- factors based on ALL data will be even larger.

### Fractional atomic coordinates and isotropic or equivalent isotropic displacement parameters (Å^2^)

**Table:**

	*x*	*y*	*z*	*U*_iso_*/*U*_eq_	
S1	0.77443 (3)	0.08125 (4)	0.20862 (3)	0.02569 (10)	
O1	0.41530 (10)	−0.09376 (9)	0.16072 (9)	0.02482 (19)	
O2	0.49565 (11)	−0.32309 (10)	0.42671 (10)	0.0303 (2)	
O3	0.62246 (13)	−0.48943 (11)	0.33781 (12)	0.0398 (3)	
H3O	0.583 (2)	−0.549 (2)	0.414 (2)	0.063 (6)*	
C3	0.43798 (13)	0.27862 (13)	0.16349 (12)	0.0225 (2)	
H3	0.5125	0.3395	0.1697	0.027*	
C1	0.60123 (13)	0.03588 (13)	0.18486 (12)	0.0219 (2)	
C2	0.47208 (13)	0.13333 (13)	0.16428 (12)	0.0211 (2)	
C4	0.29330 (13)	0.33348 (13)	0.15341 (12)	0.0226 (2)	
C5	0.18669 (14)	0.24291 (14)	0.13832 (13)	0.0252 (3)	
H5	0.0891	0.2821	0.1288	0.030*	
C6	0.21845 (14)	0.09865 (14)	0.13680 (13)	0.0253 (3)	
H6	0.1458	0.0387	0.1260	0.030*	
C7	0.36164 (14)	0.04727 (13)	0.15196 (12)	0.0219 (2)	
C8	0.55972 (13)	−0.09689 (14)	0.18353 (12)	0.0236 (3)	
C9	0.24973 (13)	0.48216 (13)	0.17527 (13)	0.0230 (2)	
H9	0.3408	0.5437	0.1451	0.028*	
C10	0.20154 (14)	0.45933 (14)	0.32704 (13)	0.0262 (3)	
H10A	0.1163	0.3920	0.3607	0.031*	
H10B	0.2847	0.4119	0.3747	0.031*	
C11	0.15678 (15)	0.60348 (15)	0.35951 (14)	0.0289 (3)	
H11A	0.1191	0.5821	0.4573	0.035*	
H11B	0.2454	0.6657	0.3374	0.035*	
C12	0.03734 (15)	0.68695 (15)	0.28067 (15)	0.0316 (3)	
H12A	0.0154	0.7828	0.2992	0.038*	
H12B	−0.0553	0.6297	0.3100	0.038*	
C13	0.08862 (16)	0.71312 (14)	0.13001 (14)	0.0303 (3)	
H13A	0.1767	0.7769	0.0995	0.036*	
H13B	0.0083	0.7649	0.0804	0.036*	
C14	0.12848 (16)	0.56783 (14)	0.09797 (14)	0.0289 (3)	
H14A	0.1637	0.5882	−0.0001	0.035*	
H14B	0.0384	0.5072	0.1223	0.035*	
C15	0.63734 (15)	−0.24245 (14)	0.20305 (14)	0.0274 (3)	
H15A	0.7446	−0.2299	0.2007	0.033*	
H15B	0.6281	−0.2804	0.1274	0.033*	
C16	0.57635 (14)	−0.35434 (13)	0.33429 (13)	0.0247 (3)	
C17	0.73084 (15)	0.08854 (16)	0.38308 (14)	0.0292 (3)	
H17	0.6358	0.1463	0.3937	0.035*	
C18	0.7115 (2)	−0.06369 (19)	0.48197 (15)	0.0445 (4)	
H18A	0.8041	−0.1215	0.4727	0.067*	
H18B	0.6303	−0.1124	0.4635	0.067*	
H18C	0.6876	−0.0562	0.5740	0.067*	
C19	0.85552 (19)	0.1697 (2)	0.40621 (18)	0.0453 (4)	
H19A	0.8377	0.1720	0.4999	0.068*	
H19B	0.8588	0.2707	0.3453	0.068*	
H19C	0.9507	0.1188	0.3884	0.068*	

### Atomic displacement parameters (Å^2^)

**Table:**

	*U*^11^	*U*^22^	*U*^33^	*U*^12^	*U*^13^	*U*^23^
S1	0.02160 (15)	0.02993 (17)	0.02262 (16)	−0.00101 (11)	−0.00132 (11)	−0.00472 (13)
O1	0.0289 (4)	0.0194 (4)	0.0266 (5)	0.0012 (3)	−0.0066 (4)	−0.0067 (4)
O2	0.0376 (5)	0.0218 (4)	0.0271 (5)	0.0013 (4)	0.0008 (4)	−0.0046 (4)
O3	0.0553 (7)	0.0199 (5)	0.0346 (6)	0.0067 (4)	0.0054 (5)	−0.0031 (4)
C3	0.0246 (6)	0.0209 (6)	0.0220 (6)	−0.0014 (4)	−0.0042 (5)	−0.0061 (5)
C1	0.0229 (5)	0.0227 (6)	0.0177 (5)	0.0006 (4)	−0.0021 (4)	−0.0034 (5)
C2	0.0234 (5)	0.0223 (6)	0.0164 (5)	−0.0001 (4)	−0.0029 (4)	−0.0043 (5)
C4	0.0263 (6)	0.0207 (5)	0.0201 (6)	0.0006 (4)	−0.0032 (5)	−0.0057 (5)
C5	0.0236 (6)	0.0256 (6)	0.0275 (6)	0.0021 (4)	−0.0060 (5)	−0.0092 (5)
C6	0.0269 (6)	0.0241 (6)	0.0266 (6)	−0.0018 (5)	−0.0069 (5)	−0.0084 (5)
C7	0.0277 (6)	0.0190 (5)	0.0188 (5)	0.0007 (4)	−0.0041 (5)	−0.0053 (5)
C8	0.0256 (6)	0.0235 (6)	0.0188 (6)	0.0020 (4)	−0.0023 (5)	−0.0034 (5)
C9	0.0249 (6)	0.0198 (5)	0.0253 (6)	0.0008 (4)	−0.0051 (5)	−0.0078 (5)
C10	0.0309 (6)	0.0239 (6)	0.0252 (6)	0.0040 (5)	−0.0092 (5)	−0.0077 (5)
C11	0.0343 (7)	0.0277 (6)	0.0271 (7)	0.0024 (5)	−0.0063 (5)	−0.0116 (6)
C12	0.0324 (7)	0.0275 (6)	0.0345 (7)	0.0068 (5)	−0.0042 (6)	−0.0111 (6)
C13	0.0365 (7)	0.0225 (6)	0.0313 (7)	0.0070 (5)	−0.0105 (6)	−0.0060 (5)
C14	0.0375 (7)	0.0241 (6)	0.0267 (6)	0.0049 (5)	−0.0120 (5)	−0.0072 (5)
C15	0.0307 (6)	0.0216 (6)	0.0260 (6)	0.0036 (5)	−0.0015 (5)	−0.0040 (5)
C16	0.0255 (6)	0.0206 (6)	0.0273 (6)	0.0018 (4)	−0.0071 (5)	−0.0049 (5)
C17	0.0280 (6)	0.0347 (7)	0.0277 (7)	−0.0011 (5)	−0.0017 (5)	−0.0148 (6)
C18	0.0580 (10)	0.0496 (9)	0.0229 (7)	−0.0175 (8)	−0.0037 (7)	−0.0045 (7)
C19	0.0461 (9)	0.0517 (10)	0.0461 (9)	−0.0131 (7)	−0.0070 (7)	−0.0239 (8)

### Geometric parameters (Å, °)

**Table:**

S1—C1	1.7488 (12)	C10—H10B	0.9900
S1—C17	1.8351 (14)	C11—C12	1.5182 (18)
O1—C7	1.3774 (14)	C11—H11A	0.9900
O1—C8	1.3781 (14)	C11—H11B	0.9900
O2—C16	1.2128 (16)	C12—C13	1.523 (2)
O3—C16	1.3160 (15)	C12—H12A	0.9900
O3—H3O	0.87 (2)	C12—H12B	0.9900
C3—C4	1.3934 (16)	C13—C14	1.5268 (18)
C3—C2	1.3942 (17)	C13—H13A	0.9900
C3—H3	0.9500	C13—H13B	0.9900
C1—C8	1.3525 (17)	C14—H14A	0.9900
C1—C2	1.4505 (16)	C14—H14B	0.9900
C2—C7	1.3932 (17)	C15—C16	1.5092 (18)
C4—C5	1.4077 (17)	C15—H15A	0.9900
C4—C9	1.5173 (16)	C15—H15B	0.9900
C5—C6	1.3882 (17)	C17—C18	1.508 (2)
C5—H5	0.9500	C17—C19	1.5160 (19)
C6—C7	1.3800 (17)	C17—H17	1.0000
C6—H6	0.9500	C18—H18A	0.9800
C8—C15	1.4832 (17)	C18—H18B	0.9800
C9—C14	1.5292 (17)	C18—H18C	0.9800
C9—C10	1.5355 (18)	C19—H19A	0.9800
C9—H9	1.0000	C19—H19B	0.9800
C10—C11	1.5250 (18)	C19—H19C	0.9800
C10—H10A	0.9900		
			
C1—S1—C17	100.95 (6)	C11—C12—C13	110.51 (11)
C7—O1—C8	105.84 (9)	C11—C12—H12A	109.5
C16—O3—H3O	108.6 (14)	C13—C12—H12A	109.5
C4—C3—C2	119.24 (11)	C11—C12—H12B	109.5
C4—C3—H3	120.4	C13—C12—H12B	109.5
C2—C3—H3	120.4	H12A—C12—H12B	108.1
C8—C1—C2	106.07 (11)	C12—C13—C14	111.07 (11)
C8—C1—S1	127.22 (10)	C12—C13—H13A	109.4
C2—C1—S1	126.70 (9)	C14—C13—H13A	109.4
C7—C2—C3	119.29 (11)	C12—C13—H13B	109.4
C7—C2—C1	105.52 (11)	C14—C13—H13B	109.4
C3—C2—C1	135.04 (11)	H13A—C13—H13B	108.0
C3—C4—C5	119.17 (11)	C13—C14—C9	111.25 (11)
C3—C4—C9	119.17 (11)	C13—C14—H14A	109.4
C5—C4—C9	121.27 (11)	C9—C14—H14A	109.4
C6—C5—C4	122.64 (11)	C13—C14—H14B	109.4
C6—C5—H5	118.7	C9—C14—H14B	109.4
C4—C5—H5	118.7	H14A—C14—H14B	108.0
C7—C6—C5	116.17 (11)	C8—C15—C16	112.95 (11)
C7—C6—H6	121.9	C8—C15—H15A	109.0
C5—C6—H6	121.9	C16—C15—H15A	109.0
O1—C7—C6	126.11 (11)	C8—C15—H15B	109.0
O1—C7—C2	110.44 (10)	C16—C15—H15B	109.0
C6—C7—C2	123.43 (11)	H15A—C15—H15B	107.8
C1—C8—O1	112.09 (11)	O2—C16—O3	123.83 (12)
C1—C8—C15	132.97 (12)	O2—C16—C15	123.84 (11)
O1—C8—C15	114.94 (11)	O3—C16—C15	112.32 (11)
C4—C9—C14	115.11 (10)	C18—C17—C19	112.04 (13)
C4—C9—C10	108.57 (10)	C18—C17—S1	111.38 (10)
C14—C9—C10	110.10 (10)	C19—C17—S1	107.38 (10)
C4—C9—H9	107.6	C18—C17—H17	108.6
C14—C9—H9	107.6	C19—C17—H17	108.6
C10—C9—H9	107.6	S1—C17—H17	108.6
C11—C10—C9	112.52 (10)	C17—C18—H18A	109.5
C11—C10—H10A	109.1	C17—C18—H18B	109.5
C9—C10—H10A	109.1	H18A—C18—H18B	109.5
C11—C10—H10B	109.1	C17—C18—H18C	109.5
C9—C10—H10B	109.1	H18A—C18—H18C	109.5
H10A—C10—H10B	107.8	H18B—C18—H18C	109.5
C12—C11—C10	111.41 (11)	C17—C19—H19A	109.5
C12—C11—H11A	109.3	C17—C19—H19B	109.5
C10—C11—H11A	109.3	H19A—C19—H19B	109.5
C12—C11—H11B	109.3	C17—C19—H19C	109.5
C10—C11—H11B	109.3	H19A—C19—H19C	109.5
H11A—C11—H11B	108.0	H19B—C19—H19C	109.5
			
C17—S1—C1—C8	−104.25 (12)	C2—C1—C8—C15	−177.78 (13)
C17—S1—C1—C2	74.88 (12)	S1—C1—C8—C15	1.5 (2)
C4—C3—C2—C7	1.42 (18)	C7—O1—C8—C1	−2.16 (14)
C4—C3—C2—C1	−173.37 (13)	C7—O1—C8—C15	177.18 (10)
C8—C1—C2—C7	−0.07 (14)	C3—C4—C9—C14	150.58 (12)
S1—C1—C2—C7	−179.35 (9)	C5—C4—C9—C14	−36.65 (16)
C8—C1—C2—C3	175.22 (13)	C3—C4—C9—C10	−85.52 (13)
S1—C1—C2—C3	−4.1 (2)	C5—C4—C9—C10	87.25 (14)
C2—C3—C4—C5	−2.77 (18)	C4—C9—C10—C11	179.46 (10)
C2—C3—C4—C9	170.16 (11)	C14—C9—C10—C11	−53.70 (14)
C3—C4—C5—C6	1.88 (19)	C9—C10—C11—C12	54.49 (14)
C9—C4—C5—C6	−170.89 (12)	C10—C11—C12—C13	−55.52 (15)
C4—C5—C6—C7	0.42 (19)	C11—C12—C13—C14	57.35 (15)
C8—O1—C7—C6	−176.30 (12)	C12—C13—C14—C9	−57.68 (15)
C8—O1—C7—C2	2.07 (13)	C4—C9—C14—C13	178.08 (11)
C5—C6—C7—O1	176.30 (11)	C10—C9—C14—C13	54.99 (14)
C5—C6—C7—C2	−1.87 (19)	C1—C8—C15—C16	109.45 (16)
C3—C2—C7—O1	−177.44 (10)	O1—C8—C15—C16	−69.71 (14)
C1—C2—C7—O1	−1.26 (14)	C8—C15—C16—O2	−14.72 (18)
C3—C2—C7—C6	0.98 (19)	C8—C15—C16—O3	166.22 (11)
C1—C2—C7—C6	177.17 (12)	C1—S1—C17—C18	73.38 (11)
C2—C1—C8—O1	1.39 (14)	C1—S1—C17—C19	−163.61 (11)
S1—C1—C8—O1	−179.34 (9)		

### Hydrogen-bond geometry (Å, °)

**Table:**

Cg1 is the centroid of the C2–C7 benzene ring.

**Table:**

*D*—H···*A*	*D*—H	H···*A*	*D*···*A*	*D*—H···*A*
O3—H3O···O2^i^	0.87 (2)	1.81 (2)	2.6745 (14)	179 (2)
C15—H15B···Cg1^ii^	0.99	2.77	3.578 (2)	140

Symmetry codes: (i) −*x*+1, −*y*−1, −*z*+1; (ii) −*x*+1, −*y*, −*z*.
